# The relationship of perivascular adipose tissue and atherosclerosis in the aorta and carotid arteries, determined by magnetic resonance imaging

**DOI:** 10.1177/1479164118757923

**Published:** 2018-02-15

**Authors:** Mohammad Alkhalil, Evan Edmond, Laurienne Edgar, Janet E Digby, Omar Omar, Matthew D Robson, Robin P Choudhury

**Affiliations:** 1 Division of Cardiovascular Medicine, Radcliffe Department of Medicine, University of Oxford and John Radcliffe Hospital, Oxford, UK; 2 Acute Vascular Imaging Centre, Radcliffe Department of Medicine, University of Oxford and John Radcliffe Hospital, Oxford, UK; 3 Centre for Statistics in Medicine, University of Oxford, Oxford, UK

**Keywords:** Perivascular adipose tissue, magnetic resonance imaging, atherosclerosis, carotid arteries, aorta

## Abstract

**Background and aims:**

Imaging studies have relied on the ‘overall’ volumetric quantification of perivascular adipose tissue. We sought to assess the relationship of circumferential distribution between perivascular adipose tissue and adjacent wall thickness of carotid and aortic arteries using dedicated magnetic resonance imaging sequences.

**Methods:**

Vessel wall and perivascular adipose tissue were acquired using magnetic resonance imaging (1.5 T). Co-registered images were segmented separately, and measurements of both perivascular adipose tissue and vessel wall were obtained along radii of the vessel spaced at angles of 5° each.

**Results:**

In total, 29 patients were recruited. Perivascular adipose tissue thickness of the aorta was 3.34 ± 0.79 mm with specific pattern of ‘double peaks’ distribution, while carotid perivascular adipose tissue had no identifiable pattern with thickness of 0.8 ± 0.91 mm. Although statistically significant, the correlation between perivascular adipose tissue thickness and wall thickness in carotid arteries with normal (r = 0.040, *p* = 0.001) or with abnormal wall thickness (r = –0.039, *p* = 0.015) was merely nominal. Similarly, perivascular adipose tissue of the aorta had very weak correlation with normal aortic wall thickness (r = 0.010, *p* = 0.008) but not with the abnormal ones (r = −0.05, *p* = 0.29).

**Conclusion:**

Dissociation between the spatial distribution of perivascular adipose tissue and arterial wall thickening in the aorta and carotid arteries does not support that perivascular adipose tissue has a causal role in promoting atherosclerotic plaque via a paracrine route. Yet, perivascular adipose tissue functional properties were not examined in this study.

## Introduction

Adipose tissue has been increasingly recognised as an active endocrine organ that not only responds to afferent signals via numerous receptors but also secretes a variety of adipokines.^1^ The considerable differences in secreted adipokines characterise adipose tissue and contribute to its anatomical regional heterogeneity.^2^ While the association between visceral adiposity, for example, and the proinflammatory status has been proposed,^3^ the role of perivascular adipose tissue (PVAT), however, has been less clear. PVAT-derived adiponectin has been shown to have a vasoprotective impact through its vasodilatory properties and stimulation of endothelial nitric oxide synthase (eNOS).^4,5^ However, a contrary role for PVAT as atherogenic is supported by studies in obese humans and mice, where hypertrophy of adipocytes result in the migration of monocytes via the release of monocyte chemoattractant protein-1 (MCP-1).^2,6–9^ Subsequently, the production of proinflammatory cytokines such as tumour necrosis factor-α (TNF-α) in differentiated monocytes leads to direct inhibition of insulin signalling in adipocytes.^2,6–9^

While there is a lack of consensus on whether PVAT has a protective or pro-atherosclerotic role, there is more evidence to suggest that PVAT is locally regulated and exerts its effect via paracrine adipokine signalling.^10,11^ The anatomical proximity of PVAT and vessel wall and the evidence of their connectivity through microvessels have fuelled the arguments of potential cross-talk and possibility that PVAT may be involved in the initiation and progression of atherosclerosis.^12^ The direct interaction between PVAT with the outer adventitia has suggested the ability to convey adipokines to vessel wall via the vasa vasorum.^13,14^ Moreover, the absence of atherosclerosis in segments of ‘myocardial bridging’ where heart muscle insulates a coronary artery from any adipose tissue has also favoured the argument for PVAT role in the development of vascular disease.^12^

Magnetic resonance imaging (MRI) has been used to great effect for the high-resolution characterisation of carotid arteries and aorta.^15–17^ Differential suppression techniques can be used to isolate signals from fat or water, which separates PVAT (by suppressing water signal) and vessel wall (by suppressing fat signal) accurately, thus overcoming the difficulty of the anatomical proximity. Accordingly, we reasoned that systematic evaluation of the spatial relationships between arterial wall thickening/atherosclerosis and adjacent PVAT with MRI may inform the contentious issue of whether PVAT has an atheroprotective or atherogenic role.

The aims of this study were to use vascular MRI (1) to study the distribution of PVAT in relation to the carotid arteries and aorta and (2) to establish whether there is a relationship between the distribution of PVAT and wall thickening/atherosclerosis.

## Patients and methods

### Study population

Patients scheduled for invasive coronary angiogram at Oxford University Hospitals NHS Trust were recruited and scanned at the University of Oxford Centre for Clinical Magnetic Resonance Research (OCMR). Patients were adult (>18 years) and were scheduled either (1) electively for assessment of symptoms suggestive of coronary artery disease and/or valvular heart disease or (2) urgent admission with acute coronary syndrome (ACS). ACS was defined as chest discomfort associated with electrocardiogram (ECG) changes and/or elevated cardiac biomarkers. Patients with known chronic inflammatory conditions, infections, malignancies or contraindications to MRI were excluded. All included patients in this study provided written informed consent. The study protocol conformed to the ethical guidelines of the Declaration of Helsinki and was approved by the National Research Ethics Services (NRES) and local R&D committee prior to commencement of the study.

### MRI protocol

Patients were imaged on a Siemens Sonata 1.5-T scanner (Siemens Healthcare, Erlangen, Germany). A black-blood turbo spin echo (TSE), fat suppressed sequence was acquired to localise carotid bifurcation, which was used as a landmark to obtain carotid images. T1-weighted TSE [repetition time (TR):echo time (TE) 700:12 ms] spanned the carotid bifurcation with 13 slices of 3 mm thickness. Slices were centralised on the identified carotid bifurcation and used to measure vessel wall thickness. Fat saturation sequence (field of view 150 × 150 mm, TR:TE 750:12 ms, in-plane spatial resolution 0.39 × 0.39 mm, water suppression) was acquired to match the obtained T1-weighted slices and suppressing signal from vessel wall producing a separated and enhanced fat image. Similarly, the descending aorta was imaged with a black-blood TSE, fat suppressed sequence to identify pulmonary bifurcation as a landmark, with 11 slices of 5 mm thickness were acquired downwards. T1-weighted TSE (TR:TE 750: 11 ms), of 11 slices of 5 mm thickness of the descending thoracic aorta, with matching fat saturated slices, were obtained.

### Data analysis

Both PVAT and vessel wall images were analysed using measurements along the radii of the vessel passing through cords on the vessel circumference. These cords were at evenly spaced out angles of 5° (starting from 0° to 355°; i.e. 72 cords per vessel image). All images were analysed by an experienced observer blinded to patient identity and background (E.E.). Analyses were performed separately for the aorta and for the carotid arteries. This process was executed using Image-Pro Plus software (Media Cybernetics, Rockville, MD).

#### PVAT image segmentation

**Figure 1.**
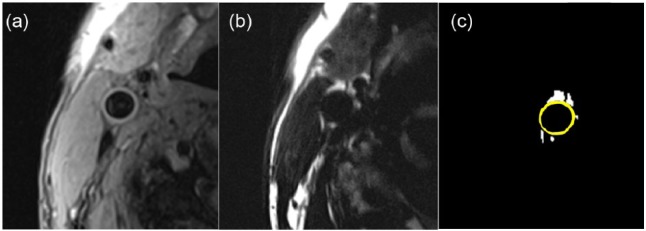
Masking effect of vessel wall and adipose tissue using Image-Pro Plus: (a) a cross-sectional T1-weighted image of a right carotid artery, (b) a matched slice of the right carotid artery using a dedicated fat saturation sequence to exhibit the PVAT and (c) the segmented PVAT using the set threshold of 2 standard deviations above the average pixel intensity values with an overlaid mask for carotid vessel wall (denoted in yellow).

Segmentation was based on a threshold intensity method, set at 2 standard deviations greater than the mean pixel intensity in the whole image (Figure 1). The user-interface requirements were to exclude the background and the very bright superficial subcutaneous tissue and to identify the centre of the vessel from where the measurements of PVAT will be obtained.

#### Measurement

There is a little consensus in the literature as to the anatomical classification of PVAT. Lehman et al.^18^ used an arbitrary box to identify PVAT when studying PVAT of the thoracic aorta. Borders of this box were the anterior border of the vertebral body, the left costovertebral joint and the oesophagus. In another study by the same group,^19^ a threshold for PVAT surrounding the abdominal aorta was set at 5 mm away from the vessel wall. In this study, we set up a distance from the outer boundary of the vessel wall to standardise our approach from patient to patient. The distance used here was derived using 2 standard deviations of the mean in measurement of PVAT made from sample images (n = 15) and resulting in a threshold at 20 mm in the aorta and 9 mm in the carotid. The intention of this method was to include the full physiological range of normal PVAT distribution, which may have been overlooked with smaller, arbitrary thresholds.

Using the Line Profile tool, the thickness measurements of both PVAT and vessel wall were made along the 72 cords around the circumference of the vessel. The centre from which these measurements were taken was defined using a software-defined best-fit circle of the vessel wall. In the carotid arteries’ slices, the zero angle position was defined as the anterior direction, and the angle was increased in a clockwise rotation. The position of the aorta relative to the spinal column varied between patients; therefore, the line drawn between the centre of the vertebral body and the centre of the aorta was used as the zero position. In order to facilitate rapid measurement, macro scripts for Image-Pro Plus were developed and used to automate part of the process.

### Defining ‘normal’ and ‘abnormal’ vessel walls

For this analysis, individual slices were categorised into ‘abnormal’ and ‘normal’ groups to allow those slices with plaque formation to be considered separately and to investigate the association of atherosclerotic plaque with PVAT. We identified a subsample of patients for both carotid and aortic wall with no eccentric or focal thickening using a modified American Heart Association (AHA) classification of vascular wall.^20^ Maximal vessel wall thickening across all angles was measured in normal subjects and a threshold value for segregating ‘normal’ from ‘abnormal’ slice was set at 3 standard deviations away from mean of maximal wall thickness. This threshold was used to ensure that only slices with evident atherosclerosis are grouped together and to avoid any observer bias in defining normal versus abnormal slices when applied to the whole cohort.

### Statistical analysis

Data were tested for normal distribution using Shapiro–Wilk test. Data were expressed as frequencies and percentages for categorical variables, mean and (±)standard deviation for continuous variables or as median accompanied by interquartile range (IQR) for skewed continuous variables, as appropriate. A test for autocorrelation was performed on all available measurements included in the analysis, which were taken at 5° angles and showed strong autocorrelation. Measurements at angles just 5° apart contain too much similar information to measurements taken at neighbouring angles, so inclusion of them all would inappropriately inflate the apparent significance of the results. Autocorrelation was overcome by including only angles 20° apart in the analysis. Wall thickness was regressed on PVAT thickness using hierarchical regression analysis, with random components for between-person variance, angle variance and slice variance. In addition, angle and slice were fitted as categorical predictor variables to take account of any systematic patterns in these variables across individuals. The modelled association between wall thickness and PVAT thickness is, therefore, in person-specific variance over and above the component of the pattern by angle and slice that are common across all people. Analyses were then repeated, with normal and abnormal slices analysed separately, to find the associations in the presence and in the absence of atherosclerosis. Results were reported in terms of regression coefficients, with confidence intervals (CIs), which were derived from hierarchical regression results. Analyses were performed using MLwiN software version 2.10.^21^

## Results

A total of 29 patients were recruited into the study. One patient had poor quality MRI for both aorta and carotid arteries and was excluded from the image analysis. The remaining patients yielded 1180 of combined carotid and aortic slices resulting in 84,960 adjoining segments have been analysed.

The mean age was 68 ± 8 years, and 68% of the cohort was male. Baseline patient clinical characteristics are summarised in Table 1. Most subjects (72%) were elective admissions for symptoms suggestive of coronary artery disease. The recruited patients represented a heterogeneous cohort ranged from no significant coronary disease (39%) to severe triple vessel disease requiring bypass surgery in 29%.

**Table 1.** Clinical characteristics of recruited patients.

Baseline characteristics		Total cohort (N = 28)
Age (years), mean ± SD		68 ± 8
Male gender (%)		19 (68%)
BMI (kg/m^2^), mean ± SD		27.2 ± 3.4
Hypertension (%)		21 (75%)
Diabetes mellitus (%)		6 (21%)
Smoking (%)		8 (29%)
Family history of CVD (%)		11 (39%)
Medications (%)	Antiplatelet	20 (71%)
	Beta blockers	17 (61%)
	Calcium channel blockers	5 (18%)
	ACE/ARB	19 (68%)
	Statin	21 (75%)
	Anticoagulation	4 (14%)
	Insulin	2 (7%)
HDL cholesterol (mmol/L), mean ± SD		1.3 ± 0.3
LDL cholesterol (mmol/L), mean ± SD		2.7 ± 0.8
Triglycerides (mmol/L), mean ± SD		1.3 ± 0.5
Total cholesterol (mmol/L), mean ± SD		4.6 ± 1.0
Indication for CA	Symptom assessment	20 (72%)
	Hospital admission for ACS	4 (14%)
	Work up for valve surgery	4 (14%)
Status of coronary arteries	Unobstructed coronary arteries	11 (39%)
	Single-vessel disease	5 (18%)
	Two-vessel disease	4 (14%)
	Three-vessel disease	8 (29%)

BMI: body mass index; SD: standard deviation; ACE: angiotensin converting enzyme; ARB: angiotensin receptor blockers; CA: coronary angiogram; HDL: high-density lipoprotein; LDL: low-density lipoprotein; CVD: cardiovascular disease; ACS: acute coronary syndrome.

PVAT and vessel wall thickness was studied in aorta with an average of 10.86 ± 0.44 slices per patient and in both left and right carotid arteries yielding an average of 10.2 ± 1.54 common carotid slices, 2.76 ± 1.54 internal carotid and 2.76 ± 1.54 external carotid slices per patient.

PVAT slices (total of >50) from 10 randomly selected patients were re-analysed yielding an inter-observer coefficient of variation of 6.12%.

### Distribution of PVAT and wall thickness

**Figure 2.**
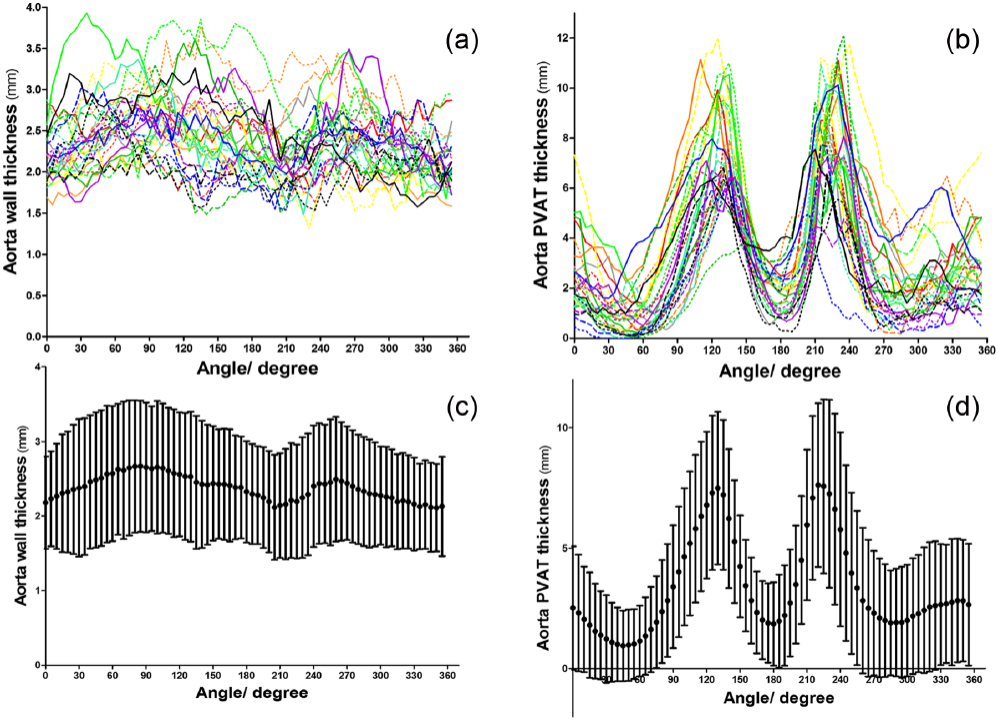
Spatial and circumferential distribution of PVAT and vessel wall thickness of aorta: (a) the individual variation of vessel wall thickness of all recruited patients, assigning every patient into different colour and/or different pattern, (b) the individual distribution of PVAT with the ‘double peaks’ phenomenon appears consistent across most patients, (c) the changes in average mean aortic wall thickness with standard deviations of all patients across different angles and (d) the average PVAT thickness of all patients’ aorta with ‘M’ shape at angles 120° and 220°.

The mean wall thickness of the aorta was 2.38 ± 0.79 mm with average PVAT thickness of 3.34 ± 0.79 mm. Changes in both aortic wall and PVAT thickness across angles among individual patients are presented in Figure 2(a) and (b). The changes in the *mean* aortic wall thickness across different angles were largely uniform (IQR: 2.24–2.49 mm; Figure 2(c)), while the *mean* PVAT thickness appeared to show wider variations (IQR: 1.96–4.54 mm). Interestingly, the mean PVAT thickness appeared to show a distinctive pattern of ‘M’ or ‘double peak’ shape across all patients with maximal peaks at angles 120° and 220°. The dispersion of PVAT thickness was larger than the aortic vessel wall thickness (coefficients of variation 0.90 vs 0.32, *p* < 0.001).

**Figure 3.**
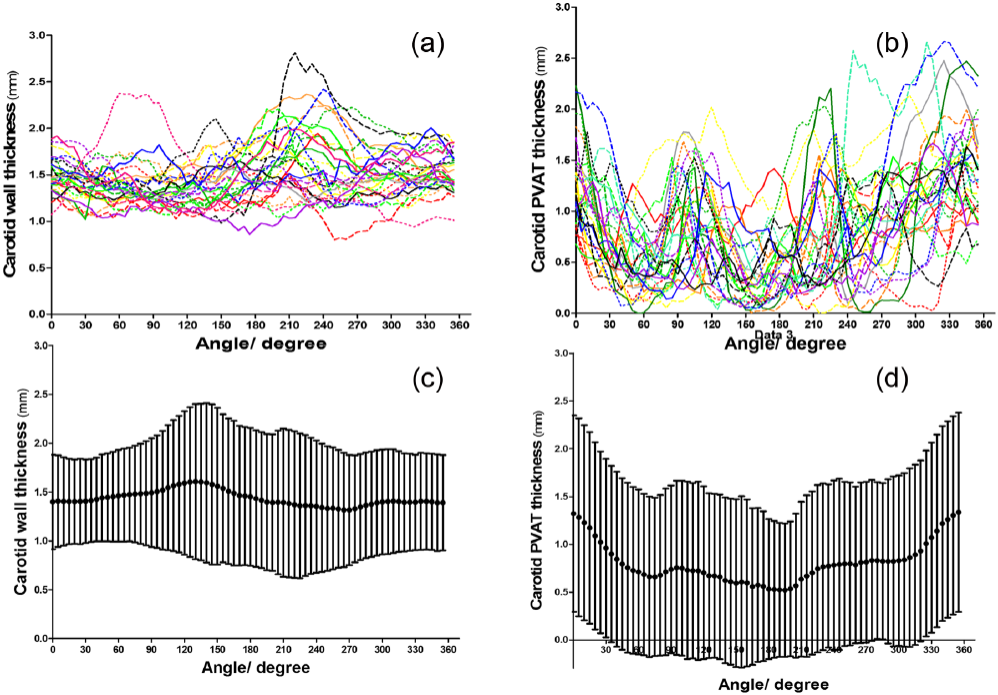
Spatial and circumferential distribution of PVAT and vessel wall thickness of the right carotid arteries: (a) the individual variation of vessel wall thickness of all recruited patients, assigning every patient into different colours and/or different patterns; (b) the individual distribution of PVAT with the very irregular shapes compared to the aorta; (c) the changes in average mean carotid wall thickness with standard deviations of all patients across different angles; and (d) the average PVAT thickness of all patients’ right carotid arteries with maximal thickness between 325° and 25°.

The mean carotid wall thickness was 1.4 ± 0.6 mm with average carotid PVAT thickness of 0.8 ± 0.91 mm. Changes in both carotid wall and PVAT thickness across angles among individual patients are presented in Figure 3(a) and (b). The changes in the *mean* carotid wall thickness across angles was largely uniform (IQR: 1.39−1.48 mm; Figure 3(c)), while the *mean* PVAT thickness appeared to show wider variations (IQR: 0.66–0.85 mm). There was no identifiable pattern of PVAT distribution, but carotid PVAT appeared to be thicker between angles 325°−360º and 0°−25°. The dispersion of carotid PVAT thickness was larger than carotid vessel wall thickness (coefficients of variation 1.15 vs 0.42, *p* < 0.001).

### Association between PVAT and wall thickness

There was a statistically significant but very weak positive correlation between normal carotid vessel wall and PVAT thickness [r = 0.040 (0.016–0.065), *p* = 0.001], whereas a negative correlation between abnormal carotid and PVAT thickness [r = −0.039 (−0.070 to −0.008), *p* = 0.015; Table 2].

**Table 2.** Hierarchical regression analysis of vessel wall thickness and PVAT.

	Correlation	95% Confidence interval	*p*-value
Carotid (normal)	R = 0.040	0.016 to 0.065	0.001
Carotid (abnormal)	R = −0.039	−0.070 to −0.008	0.015
Aorta (normal)	R = 0.010	0.003 to 0.017	0.008
Aorta (abnormal)	R = −0.050	−0.142 to 0.043	0.29

PVAT: perivascular adipose tissue.

Normal aortic wall had a positive association with PVAT thickness [r = 0.01 (0.003–0.017), *p* = 0.008], while abnormal aortic wall did not correlate with PVAT thickness [r = −0.050 (−0.142 to 0.043), *p* = 0.29].

When excluding patients presenting with ACS, the nominal relationship between PVAT and both aortic and carotid wall thickness did not change for both normal and abnormal wall thickness. Similarly, when excluding patients with non-obstructive coronary artery disease, the relationship between PVAT and carotid wall thickness was maintained in both normal (0.015, 95% CI: 0.003–0.026, *p* = 0.012) and abnormal carotid wall thickness (−0.066, 95% CI: −0.131 to −0.002, *p* = 0.045). Similarly, the relationship between PVAT and aortic wall thickness followed the same trend of significance with normal (0.011, 95% CI: 0.002–0.020, *p* = 0.018) and abnormal aortic wall thickness (−0.556, 95% CI: −1.589 to 0.477, *p* = 0.25). When interaction terms between angle and slice were included in the analysis, the obtained results were very similar (results not shown).

## Discussion

Using a newly devised methodology to quantify MRI-identified PVAT, we have demonstrated that (1) PVAT is more variable than vessel wall thickness among patients and across angular distribution; (2) aortic PVAT follows a similar pattern between individuals, while carotid PVAT is less consistent; and (3) there was no biologically meaningful correlation in angular distribution between PVAT and atherosclerosis in either aorta or carotid arteries, irrespective of the burden of atherosclerosis.

While the role of PVAT in promoting atherosclerosis by secreting pro-atherogenic factors has been widely considered,^12^ there is also a significant body of contradictory evidence to suggest that it has a protective role.^4,22,23^ Numerous factors related to PVAT, vascular location and the physiological status of PVAT might contribute to this apparent paradox. The variations of adipocyte composition, stage of differentiation and morphology with subsequent different local effect add to the discrepancy in the role of PVAT.^10,24^ Moreover, the anatomical regional vascular differences have been reported to influence the role of PVAT.^10,24^ Therefore, any broad statement on the selective and predefined role of PVAT, whether it is pro-atherogenic or vascular protective, is likely to be overly simplistic.

Imaging has added insights into the atherosclerotic role of PVAT.^18,25^ The increase in PVAT measured on computed tomography (CT), using a distance-based method of the sum of volume of Hounsfield unit-defined adipose tissue in a 5-mm increments along the coronary artery, was associated with the severity of coronary artery disease and features of high-risk atherosclerotic plaques.^10,26,27^ In this study, using MRI-driven methodology, the distribution and association between PVAT and vessel wall thickness were investigated. We used wall thickness to reflect the atherosclerosis burden, where thick segments represented more advanced disease. We demonstrated that PVAT distribution is widely irregular in comparison to vessel wall thickness irrespective of the degree of wall thickening. We also showed that aortic PVAT followed a characteristic pattern with two prominent peaks within the posterior region of the aortic wall. A similar pattern was not identified in the carotid artery, but PVAT tended to be thicker on the anterior surface (angle 325°–25°) compared to other angles. We detected very weak correlation between PVAT and adjacent wall thickness in both aorta and carotid arteries and irrespective of the plaque burden. The correlation coefficient across all groups was very close to 0 (ranging between −0.05 and 0.04), suggesting that there was no strongly determinant inter-relationship. The *p*-value in the ‘abnormal’ aortic slices group did not reach statistical significance and is probably a reflective of smaller sample size compared to other categories. These findings do not support the hypothesis of the atherosclerotic role of PVAT via paracrine local effect. Nonetheless, it is important to highlight that the current analysis did not investigate the functional properties of PVAT nor its possible impact on vessel wall composition.

In vitro experiments using human periaortic adipocytes have shown their ability to stimulate migration of inflammatory cells. In addition, in apolipoprotein E knock-out mice, there was an increase in infiltrating macrophage within PVAT and similarly an increase in PVAT release of proinflammatory cytokines.^28,29^ Similarly, in humans, macrophage infiltration and adipokines production were different comparing PVAT near stenotic and non-stenotic coronary artery segments.^30^ Furthermore, imaging studies have demonstrated an association between the burden of atherosclerosis and increase PVAT volume.^10,25–27^ However, the proposed pro-atherogenic role could be challenged given the cross-sectional design of these studies, which do not provide a causal relationship between PVAT and atherosclerosis.^18,25^ A reverse relationship could be proposed where atherosclerosis and vessel wall inflammation may lead into increase in PVAT (inside–out theory) and not vice versa.^12^ Previous studies have relied on the total volumetric quantity of PVAT (i.e. ignoring adjacencies) to relate it to atherosclerosis via a local paracrine influence.^10^ Adipokines secreted from PVAT pass through vasa vasorum or through adventitia and media to reach vessel intima. We did not identify any relationship between the spatial distribution of PVAT and atherosclerosis measured as vessel wall thickness; however, few points need to be highlighted. First, the simultaneous measurements of PVAT and vessel wall thickness may be challenged given the snapshot analysis of a heterogonous population. Second, while there may still be an influence of PVAT on atherosclerosis, its impact may not be sufficient to show anatomical changes on the vascular wall as we examined here. Third, the influence of PVAT may be subtle, for example, on plaque composition or inflammatory cell infiltrate. Finally, it is arguable that quantification of PVAT may not play a significant role but rather the change in its adipocytes character, structure and morphology is more biologically important. In a post mortem study of 16 patients, coronary atherosclerotic burden expressed as plaque/media ratio was related to PVAT area and degree of macrophage infiltration within PVAT.^26^ In addition, PVAT was correlated to the presence of lipid core.^26^ This assessment was made again based on a measured area of 3-mm circle around the studied coronary vessel.^26^

This study adds to the body of evidence examining the possible role of PVAT in atherogenesis. Most reported studies have focused on coronary arteries and to less extent aorta. Our study provides for the first time a systematic detailed description of PVAT of the carotid arteries in addition to the aorta. While others have used CT to study PVAT, we have demonstrated the utility of MRI as an alternative modality. We demonstrated lack of relationship between the angular distribution of PVAT and arterial wall thickness in the aorta and carotid arteries. Such dissociation does not support the concept that PVAT has a causal role in promoting atherosclerosis.

## References

[1] Kershaw EE Flier JS. Adipose tissue as an endocrine organ. J Clin Endocrinol Metab 2004; 89: 2548–2556. 15181022 10.1210/jc.2004-0395

[2] Fitzgibbons TP Czech MP. Epicardial and perivascular adipose tissues and their influence on cardiovascular disease: basic mechanisms and clinical associations. J Am Heart Assoc 2014; 3: e000582. 24595191 10.1161/JAHA.113.000582 PMC4187500

[3] Van Gaal LF Mertens IL De Block CE. Mechanisms linking obesity with cardiovascular disease. Nature 2006; 444: 875–880. 17167476 10.1038/nature05487

[4] Margaritis M Antonopoulos AS Digby J et al Interactions between vascular wall and perivascular adipose tissue reveal novel roles for adiponectin in the regulation of endothelial nitric oxide synthase function in human vessels. Circulation 2013; 127: 2209–2221. 23625959 10.1161/CIRCULATIONAHA.112.001133

[5] Greenstein AS Khavandi K Withers SB et al Local inflammation and hypoxia abolish the protective anticontractile properties of perivascular fat in obese patients. Circulation 2009; 119: 1661–1670. 19289637 10.1161/CIRCULATIONAHA.108.821181

[6] Gustafson B Hammarstedt A Andersson CX et al Inflamed adipose tissue: a culprit underlying the metabolic syndrome and atherosclerosis. Arterioscler Thromb Vasc Biol 2007; 27: 2276–2283. 17823366 10.1161/ATVBAHA.107.147835

[7] Hotamisligil GS Arner P Caro JF et al Increased adipose tissue expression of tumor necrosis factor-alpha in human obesity and insulin resistance. J Clin Invest 1995; 95: 2409–2415. 7738205 10.1172/JCI117936 PMC295872

[8] Baker AR Silva NF Quinn DW et al Human epicardial adipose tissue expresses a pathogenic profile of adipocytokines in patients with cardiovascular disease. Cardiovasc Diabetol 2006; 5: 1. 16412224 10.1186/1475-2840-5-1 PMC1352345

[9] Xu H Barnes GT Yang Q et al Chronic inflammation in fat plays a crucial role in the development of obesity-related insulin resistance. J Clin Invest 2003; 112: 1821–1830. 14679177 10.1172/JCI19451 PMC296998

[10] Fernandez-Alfonso MS Gil-Ortega M Aranguez I et al Role of PVAT in coronary atherosclerosis and vein graft patency: friend or foe? Br J Pharmacol 2017; 174: 3561–3572. 28150299 10.1111/bph.13734 PMC5610150

[11] Lee HY Despres JP Koh KK. Perivascular adipose tissue in the pathogenesis of cardiovascular disease. Atherosclerosis 2013; 230: 177–184. 24075741 10.1016/j.atherosclerosis.2013.07.037

[12] Verhagen SN Visseren FL. Perivascular adipose tissue as a cause of atherosclerosis. Atherosclerosis 2011; 214: 3–10. 20646709 10.1016/j.atherosclerosis.2010.05.034

[13] Xu J Lu X Shi GP. Vasa vasorum in atherosclerosis and clinical significance. Int J Mol Sci 2015; 16: 11574–11608. 26006236 10.3390/ijms160511574 PMC4463718

[14] Chatterjee TK Stoll LL Denning GM et al Proinflammatory phenotype of perivascular adipocytes: influence of high-fat feeding. Circ Res 2009; 104: 541–549. 19122178 10.1161/CIRCRESAHA.108.182998 PMC2742882

[15] Kylintireas I Shirodaria C Lee JM et al Multimodal cardiovascular magnetic resonance quantifies regional variation in vascular structure and function in patients with coronary artery disease: relationships with coronary disease severity. J Cardiovasc Magn R 2011; 13: 61. 10.1186/1532-429X-13-61 PMC3256113 22017860

[16] Alkhalil M Biasiolli L Chai JT et al Quantification of carotid plaque lipid content with magnetic resonance T2 mapping in patients undergoing carotid endarterectomy. PLoS ONE 2017; 12: e0181668. 28746385 10.1371/journal.pone.0181668 PMC5528883

[17] Alkhalil M Chai JT Choudhury RP. Plaque imaging to refine indications for emerging lipid-lowering drugs. Eur Heart J Cardiovasc Pharmacother 2017; 3: 58–67. 27816944 10.1093/ehjcvp/pvw034 PMC5841877

[18] Lehman SJ Massaro JM Schlett CL et al Peri-aortic fat, cardiovascular disease risk factors, and aortic calcification: the Framingham heart study. Atherosclerosis 2010; 210: 656–661. 20152980 10.1016/j.atherosclerosis.2010.01.007 PMC2878932

[19] Schlett CL Massaro JM Lehman SJ et al Novel measurements of periaortic adipose tissue in comparison to anthropometric measures of obesity, and abdominal adipose tissue. Int J Obes 2009; 33: 226–232. 10.1038/ijo.2008.267 PMC3779879 19139753

[20] Cai JM Hatsukami TS Ferguson MS et al Classification of human carotid atherosclerotic lesions with in vivo multicontrast magnetic resonance imaging. Circulation 2002; 106: 1368–1373. 12221054 10.1161/01.cir.0000028591.44554.f9

[21] Rasbash J Charlton C Browne WJ et al MLwiN version 2.10. Bristol: Centre for Multilevel Modelling, University of Bristol, 2009.

[22] Souza DS Johansson B Bojo L et al Harvesting the saphenous vein with surrounding tissue for CABG provides long-term graft patency comparable to the left internal thoracic artery: results of a randomized longitudinal trial. J Thorac Cardiovasc Surg 2006; 132: 373–378. 16872965 10.1016/j.jtcvs.2006.04.002

[23] Souza DS Dashwood MR Tsui JC et al Improved patency in vein grafts harvested with surrounding tissue: results of a randomized study using three harvesting techniques. Ann Thorac Surg 2002; 73: 1189–1195. 11996262 10.1016/s0003-4975(02)03425-2

[24] Gil-Ortega M Somoza B Huang Y et al Regional differences in perivascular adipose tissue impacting vascular homeostasis. Trends Endocrinol Metab 2015; 26: 367–375. 26008879 10.1016/j.tem.2015.04.003

[25] Mahabadi AA Reinsch N Lehmann N et al Association of pericoronary fat volume with atherosclerotic plaque burden in the underlying coronary artery: a segment analysis. Atherosclerosis 2010; 211: 195–199. 20223460 10.1016/j.atherosclerosis.2010.02.013

[26] Verhagen SN Vink A van der Graaf Y et al Coronary perivascular adipose tissue characteristics are related to atherosclerotic plaque size and composition. A post-mortem study. Atherosclerosis 2012; 225: 99–104. 23022141 10.1016/j.atherosclerosis.2012.08.031

[27] Maurovich-Horvat P Kallianos K Engel LC et al Influence of pericoronary adipose tissue on local coronary atherosclerosis as assessed by a novel MDCT volumetric method. Atherosclerosis 2011; 219: 151–157. 21782176 10.1016/j.atherosclerosis.2011.06.049 PMC3203345

[28] Lohmann C Schafer N von Lukowicz T et al Atherosclerotic mice exhibit systemic inflammation in periadventitial and visceral adipose tissue, liver, and pancreatic islets. Atherosclerosis 2009; 207: 360–367. 19481752 10.1016/j.atherosclerosis.2009.05.004

[29] Szasz T Bomfim GF Webb RC. The influence of perivascular adipose tissue on vascular homeostasis. Vasc Health Risk Manag 2013; 9: 105–116. 23576873 10.2147/VHRM.S33760 PMC3616689

[30] Verhagen SN Buijsrogge MP Vink A et al Secretion of adipocytokines by perivascular adipose tissue near stenotic and non-stenotic coronary artery segments in patients undergoing CABG. Atherosclerosis 2014; 233: 242–247. 24529151 10.1016/j.atherosclerosis.2013.12.005

